# Case of Apoplexia Hydrocephalica

**Published:** 1806-04

**Authors:** P. Weaver

**Affiliations:** Surgeon, of Walsall


### Case *of* Apoplexia Hydrocephalica; *communicated by* Mr. P. Weaver, *Surgeon, of Walsall.*

HYDROCEPHALUS has long arrested the attention of the sons of Esculapius, and the means suggested to conquer this hydra have been as numerous as the symptoms are complicated. You, no doubt, will coincide with me in opinion, how difficult a task it is to draw a faithful portrait of this truly proteiform disease, which assumes in its various stages, as many aspects as there are hues in the camelion. We are frequently puzzled in discriminating the symptoms occurring in children from irritation, occasioned by worms, dentition, crudities in the stomach, cum aliis, from those peculiar to the primary stage of hydrocephalus, or hydrops capitis, which must be productive of much anxiety to the practitioner as well as risque to the patient, who is seldom capable of furnishing us with much information.

The subject of the present case, was a boy 3 years old, who had been indisposed some time previous to my seeing seeing him, but which was attributed by the father (who is an intelligent and well informed man) to worms. The first symptoms were nausea and vomiting, with convulsions. I ordered an emetic, afterwards a dose of calomel, which had the desired effect, by evacuating the bowels, which were constipated. My medical avocations precluded me from visiting the patient the following day; but on the third, found he had no return of the convulsions; appeared languid and inactive, often drowsy and peevish, though at intervals cheerful, and apparently free from complaints; the head was not particularly large, yet appeared heavy and burthensome, inclined more to one side than the other; the eyes watery, and affected by an exposure to light; the pupil dilated more than usual; frequent starting and screaming in his sleep, as if much terrified; at intervals delirious; likewise an increased aqueous discharge from the nose. From the above symptoms, I had not much reason to doubt the nature and reality of the disease. On the fourth day, after minutely investigating the various symptoms and probable cause, I adopted the following plan: Ordered blisters to be applied ponc aures, and one to the occiput, to be kept open with the ung. attrah; previous to which three leeches were applied to each temple; the bowels remaining costive, repeated the calomel.

5th day. Feverish towards the evening; the face much flushed, particularly the left cheek; the pulse quick; no strabismus, nor return of convulsions; the calomel had operated; ordered the following febrifuge,

℞. Aq. puræ. ℥iij. Aq. Ammon. A. ℥ß. Spt. æth. nit. ʒß. Vin. antim ʒj. Syr. sacch. ʒß. M. Coch. j. larg. 3tiis. hor. sumend.

6th day.—The child was irritable and peevish; the eyes red and heavy, with the lucis intollerantia of Cullen; moans much in its sleep, and frequently delirious; makes but little urine; the head more pendulous. The bowels continuing costive, and paucity of urine, ordered the following:

℞. Calomel. ppt. gr. vj. Pulv. digital. gr. iij. Sacchar. alb. ʒj. M. & divid. in pulv. No. vj. Mane et vesp. sumend.

7th. No particular alteration; and as the stomach retained the powders, ordered them to be taken 4tis horis, with an anodyne hora decubitu.

8th. Had made more water; the bowels moderately lax; a tolerable good night; the symptomatic fever abated; can bear the light better. Continue the medicine.

9th.

9th. The child much the same; medicine continued.

10th. Though the child continued mending, yet considered it of consequence to keep the bowels lax, and the absorbent system in action, therefore ordered the powders to be repeated, with digital gr. j. and calomel, gr. ij. singulis 3tiis. hor. sumend. Omit the mixture. Rept. haust. anod.

11th. The blister continues discharging freely; the bowels regular; urine plentiful; and the child progressively improving both in health and strength.

13th. Found my little patient restored to perfect health, excepting slight debility, consequent upon the disease, and the active means used.

The above case, though perfect in its outlines, yet, like a rough statue, requires polishing. As to purity of diction and elegance of language, I leave to those literati, who study to please and amuse, more than edify; suffice it to observe, the present case may tend to evince the propriety of using active means, and that with a steady hand, and perseverance, *nil desperandum.* Though I have no singular practice or infallible remedy to boast of, yet have the satifaction of raising an individual, who must inevitably have fallen a victim to a disease, that destroys more subjects than the dire effects of either war, pestilence, or famine; and generally evades the most judicious mode of treatment. Thus, by a happy combination of two powerful agents, digitalis and calomel, aided by topical remedies, much good has been done, but which I am persuaded neither of them, *per se,* would have effected. I have reason to believe, that if hydrocephalus was in general undertaken, previous to the second stage, it would not, in a majority of instances, evade the usual mode of treatment.

---

*For Fistulous Sinus's.*

From ample experience I have reason to recommend the following formula, and with the elastic syringe, as equal, if not superior to any other in use; and should it prove as efficacious, in the hands of other practitioners, it will be considered (though simple) as a valuable desideratum in the chirurgical practice.

℞. Aq. calcis. ℥iß. Tinct. canth. ʒiij. Tinct. opii. ʒi. M. ft. injectio.

*Feb.* 26, 1806.

CASE

